# Myelinating cells can feel disturbances in the force

**DOI:** 10.18632/oncotarget.14240

**Published:** 2016-12-27

**Authors:** Yannick Poitelon, Gustavo Della-Flora Nunes, M. Laura Feltri

**Keywords:** YAP, TAZ, Schwann cell, Neuroscience

In the vertebrate nervous systems, many axons are ensheathed along their entire length by myelin, a multilamellar structure produced by oligodendrocytes (OLs) in the brain and spinal cord and by Schwann cells (SCs) in peripheral nerves. Myelin is critical for the proper function of the nervous system and one of the most complex cell-cell interaction of the body. It allows for rapid conduction of action potentials and provides trophic support to neurons. In myelin diseases (e.g.: multiple sclerosis, leukodystrophies, Charcot-Marie-Tooth disease), dys/demyelination is often accompanied by inflammation and impaired metabolic support to axons, leading to irreversible axon damage and permanent functional loss. Although biochemical signals leading to myelination by OLs and SCs have been studied quite extensively, the importance of mechanical stimuli for the development of myelinating glial cells has only recently been described [1-6].

Accumulating evidence demonstrates that mechanical signals are as important as chemical cues to regulate cellular processes such as migration, differentiation and proliferation. Indeed, due to their organization, cells are intrinsically responsive to physical stimuli. Cellular architecture is a model of tensional integrity or “tensegrity”, stabilized by elements of tension (such as actin and intermediate filaments) and elements resisting compression (such as microtubules). Thus, a cell presents constant tension forces (mediated by cytoskeleton elements) and intermittent local compression forces (mediated by ECM and neighbouring cells). As a consequence, the cell is stabilized in the 3D space, but is also sensitive to external mechanical perturbations, which cause rapid reorganization of the cytoskeleton. Upon stimulation, mechanical forces ultimately drive biochemical changes that influence the fate or behaviour of the cell. The pathways connecting mechanical stimulation to transcriptional changes in myelinating cells have just started to be identified, *i.e*.: the linker of nucleoskeleton and cytoskeleton (LINC) complex [5] and the mechanotransducers YAP and TAZ [4, 7].

YAP and TAZ shuttle between the cytoplasm and the nucleus to play important roles in organ growth, cell differentiation, cell proliferation and survival. In the nucleus, they interact with DNA-binding transcription factors, such as TEADs, controlling gene expression. In the cytosol, YAP and TAZ are inhibited through phosphorylation by LATS1/2 kinases. Multiple signalling pathways regulate YAP and TAZ activity, including the Hippo kinase cascade, Wnt signalling, G-protein coupled receptors and metabolic pathways, as well as the intracellular force/tension generated by the rigidity of the extracellular matrix or by the actin cytoskeleton. The function of YAP and TAZ in myelinating cells, reported in Poitelon et al. 2016, indicates they are regulated by the extracellular matrix stiffness and are critical for immature SC development and myelin gene regulation [4, 8].

Immature SCs are found in peripheral nerves around E12.5. They surround bundles of axons and organize a basal lamina around them, forming ‘families’. As development progress, immature SCs send cytoplasmic processes in the bundles to selectively recognize and sort large diameter axons destined for myelination. During this initial recognition and the subsequent myelination, the stiffness of the peripheral matrix increases dramatically [3]. In YAP and TAZ SC-null mice, SCs are unable to interact with axons in bundles and cannot progress to myelination. A possible explanation is the inaptitude of SCs ablated for YAP and TAZ to sense changes in matrix stiffness or in forces applied to their basal (SC-basal lamina), apical (SC-axon) and lateral (SC-SC) surfaces. As the formation of the basal lamina is a prerequisite for the interaction of SC with axons, it is tempting to hypothesize that the basal lamina produced by SCs has a critical role for the establishment of external mechanical forces.

We demonstrate in Poitelon et al. 2016 the role of YAP and TAZ in SCs in early development [4]. While the role of YAP in OLs has started to be explored [7], the physiological mechanical forces applied to OLs in the brain are very different from those applied to SCs. Compared to mature peripheral nerves, which present a stiffness around 50kPa [3], the brain is a very soft tissue, with stiffness usually ranging from 0.1 kPa to 10 kPa. In addition, the optimal conditions for OL function *in vitro* is 6.5 kPa [6]. This could indicate that the environment is poor in physical stimuli. However, OLs express a variety of ECM receptors, most of which are essential for migration and differentiation, indicating that interaction with the ECM is important. Additionally, when OPCs reach the axon tracts, which are supposedly more rigid than the rest of the environment, they start to proliferate and then differentiate, indicating that interactions with axons could generate mechanical forces. Furthermore, mature OLs can contact numerous axons and different parts of a same axon, possibly originating tension forces.

Despite the fact that SCs and OLs are clearly mechanosensitive, the nature of forces that affect their differentiation *in vivo* remains to be established. Future works will hopefully address these questions, as well as clarify potential implications of pathogenic alterations in the stiffness of the microenvironment, for example during demyelination and tumorigenesis.

## References

[1] Jagielska A (2012). Stem Cells Dev.

[2] Rosenberg SS (2008). Proc Natl Acad Sci U S A.

[3] Urbanski MM (2016). Scientific reports.

[4] Poitelon Y (2016). Nat Neurosci.

[5] Hernandez M (2016). J Neurosci.

[6] Lourenco T (2016). Scientific reports.

[7] Shimizu T (2016). Glia.

[8] Lopez-Anido C (2015). Glia.

